# Emotional intelligence and metacognitive awareness in predicting foreign language speaking self-efficacy

**DOI:** 10.3389/fpsyg.2026.1721694

**Published:** 2026-02-19

**Authors:** Gökçen Tekin, Seda Kaya

**Affiliations:** Turkish Education Department, Faculty of Education, Bolu Abant İzzet Baysal University, Bolu, Türkiye

**Keywords:** cognitive factors, emotional intelligence, metacognitive awareness, speaking self-efficacy, teaching Turkish as a foreign language

## Abstract

Foreign language speaking proficiency is closely linked to cognitive strategies and self-regulatory processes; however, it remains unclear which aspects of individual awareness most strongly predict speaking self-efficacy. This study examines the relationship between emotional intelligence and metacognitive awareness and foreign language speaking self-efficacy. It aims to determine whether students’ metacognitive awareness and emotional intelligence affect their speaking self-efficacy. The study designed using an explanatory research design, a type of correlational research method, employed a purposive sampling method, a non-random sampling method. The study aimed to reach students who were learning Turkish as a foreign language at university, knew enough Turkish to provide the necessary data, and were continuing their education to become fluent in Turkish. 113 students studying at B1 level at Bolu Abant İzzet Baysal University in the 2024–2025 academic year participated in the study. The Emotional Intelligence Scale, Metacognitive Awareness Inventory, and Speaking Self-Efficacy Scale for Turkish as a Foreign Language Learners were administered to the participants, and multiple regression analyses were conducted on the collected data. The analysis results showed a significant and positive relationship between the students’ speaking self-efficacy and metacognitive awareness, while no statistically significant relationship was found between emotional intelligence and speaking self-efficacy. These findings highlight the crucial role of metacognitive awareness in enhancing foreign language speaking self-efficacy and provide insights for designing instructional practices that foster learners’ self-regulation in language learning contexts.

## 1 Introduction

The complex structure of speaking skills and its nature as a productive skill requires a certain accumulation of knowledge about language. Speaking skills, an action contrary to stasis (Stam, 2018), create meaning differences by using sounds. This formation requires the ability to create a perceptual response and advanced motor skills (Flege, 1997). By its nature, the use of speaking skills cannot be determined in advance. It is a rather individual process with a combination of linguistic, cognitive factors, and context (Alonso, 2018). In addition to these individual and skill-dependent factors, trying to speak in a foreign language can make the situation even more complicated.

Learning to speak in a foreign language is different from learning one’s mother tongue. Krashen (1980) states that, unlike in a native language, speaking, which we can see as a result or output in the foreign language learning process that takes place through education, cannot be taught directly, but emerges spontaneously by establishing proficiency and reducing the emotional filter. The primary reason for this is the age factor. While the Language Acquisition Device is a privileged feature that allows us to easily acquire our mother tongue at a young age, it is suggested that people no longer have access to this feature as they grow older—probably after the age of nine. In other words, acquiring a language at an early age is a situation that happens spontaneously, while learning it at a later age becomes dependent on the individual’s efforts (Fulcher, 2014). The existence of a mother tongue already encoded in the mind (Munro, 2008) and especially the passing of the critical period (Birdsong, 2009) cause the brain’s language learning capacity (Gilakjani and Ahmadi, 2011; Lenneberg, 1967; Penfield and Roberts, 1959) to decline, especially after the ages of 11–14 and cause an accented speech (Lenneberg, 1967). In this case, speaking skills that occur spontaneously at an early age can turn into learning products in later years. The language that is easily acquired at an early age will now require the individual’s performance in the learning process of any behavior or skill. For this reason, although the difficulties experienced in a conscious and effortful process and learning through education are positive in some ways, they can also bring about many problems in language learning and use (Castro and Peck, 2005; Chen and Chang, 2004; Ganschow et al., 1995; Ganschow et al., 1998; Nickel, 1971). Lowie et al. (2018) state that speaking is perhaps the most difficult skill to learn in the process of learning a foreign language. That is because speaking follows a variable process; it has many aspects such as accuracy and fluency, and it represents the language in the most obvious way. In addition, interaction with the receiver requires control over implicit meaning, adaptation to articulation, and language-specific motor skills. The time pressure on the speaker is also an additional factor.

Although formal education is more effective than natural exposure in increasing foreign language proficiency in adults (Krashen, 2002), achieving speaking proficiency in a foreign language in classroom conditions is not easy because speaking is a productive skill that challenges students’ ability to perform a task (Khatib and Maarof, 2015). Even advanced students believe that they are not sufficiently prepared to speak outside the classroom. These difficulties may arise from the quality and adequacy of speaking opportunities in the classroom. In addition, the mental processing stages in speaking are valid for both languages. Processes such as conceptualization, planning, construction, self-monitoring, and negotiation (Aleksandrzak, 2011) can make speaking skills in a foreign language even more difficult. Expecting students to communicate before they have achieved fluency in the target language can create anxiety about making mistakes even in the best students (Horwitz et al., 1986; Lisnawati et al., 2019; Piechurska-Kuciel, 2014). All of these may cause students to describe their foreign language speaking skills as difficult (Brown and Yule, 1983; Ma, 2022; Tuan and Mai, 2015). The fact that beliefs about performance status are affected by these negative conditions while trying to learn in line with a certain goal constitutes another dimension of the whole process of achieving students’ goals, which is self-efficacy. Yang (1999) claims that beliefs about language consist of metacognitive and motivational dimensions. Students will be able to meet the requirements of language learning to the extent that self-efficacy is internalized.

### 1.1 Self-efficacy and its sources

Self-efficacy is defined as the belief that a person can successfully perform a certain behavior (Bandura, 1977; Tschannen-Moran et al., 1998), the thought of how well they an perform the actions towards the goal (Oxford and Shearin, 1994); and a motivational construct (Yang, 1999). Perceived self-efficacy is a determinant of people’s thoughts, behaviors (de Vries et al., 1988), and emotional reactions they feel when faced with difficulties (Bandura, 1982). In the educational process, students’ interest, persistence, the degree of effort they spend on learning (Bandura, 1977; Tremblay et al., 1995), the goals they choose, and the use of self-regulation strategies (Lane et al., 2004; Linnenbrink and Pintrich, 2003; Pajares, 1996; Schunk, 2003) are also the main factors affecting their motivation and learning in a foreign language (Raoofi et al., 2012).

Vicarious experiences, performance successes, verbal persuasion, and physiological states are the four primary elements known as sources of self-efficacy. Performance successes are situations experienced by individuals, and positive outcomes have been shown to increase expectations, while negative outcomes have been shown to decrease expectations. Bandura (1977) argues that people’s self-learning, that is, the determinants of efficacy, is not limited to their own experiences; the experiences of others can also be a source of efficacy expectations. Verbal persuasion and physiological states are emotional stimuli. Emotional arousal refers to the feelings and physical sensations that arise in stressful and tiring situations (Bandura, 1977). People take their physiological states into account when evaluating themselves psychologically. A high level of arousal makes the person anxious and generally weakens performance (Van Dinther et al., 2011). The degree of strength of emotional reactions to the task is actually a clue about the expectation of success or failure regarding the outcome (Jabbarifar, 2011). Low levels of arousal are more likely to have an impact on achievement. Individuals may create unrealistic anxiety when they feel inadequate. Emotional arousal is a drive that triggers avoidance behavior, meaning that moderate levels of emotional arousal have an energizing function while high levels of arousal have a reinforcing function. Presenting a person with a certain level of difficulty can provide them with strengthening experiences while also eliminating defensive/avoidant behaviors (Bandura, 1977).

Based on all these, it is thought that the level of control individuals have over tasks may affect self-efficacy. The first of these controls, which can be provided from two different aspects, is the individual’s level of cognition regarding the target task. The greater the knowledge adequacy regarding the task, the easier the individual will define the task, which is directly related to the individual’s knowledge about the task, and therefore their cognition. The ability of an individual to acquire information, to control the information they acquire and to direct themselves to complete deficiencies when necessary is possible with metacognitive awareness. Therefore, one of the sources of influence is likely to be metacognitive awareness. Another aspect is how the target task makes a person feel. As will be explained in detail below, as Krashen (1980) mentioned, while the emotional filter prevents language learning in difficult tasks, it can pave the way for permanent learning in situations that the individual can cope with. In addition, emotional intelligence has the function of softening negative emotions and strengthening positive ones, thus managing the information coming from these without suppressing or exaggerating them (Mayer and Salovey, 1997). This feature enables one to tolerate and welcome emotions regardless of their nature. Below, these two concepts, which are thought to be effective in self-efficacy in speaking a foreign language and are the starting point of the research, are discussed.

### 1.2 Metacognitive awareness

Metacognition is defined as one’s own cognitive process, products, or knowledge about them (Flavell, 1976; cited in Brown, 1977) and providing the individual with the ability to control their actions and to be free from dependence on stimuli (Metcalfe, 2008); is a late-developing skill but is important to meet one’s learning demands in higher-level skills such as thinking and problem-solving (Baker and Brown, 1980). Metacognitive strategies, which are executive in nature, provide students with cognitive tools (Paris and Winograd, 2013) and enable cognition and metacognition to work together through planning, monitoring, and evaluation (Vaidya, 1999). Students can achieve the most efficient results only with the knowledge of how to work best (Wade and Reynolds, 1989). In addition, since metacognition requires the ability to introspect one’s performance and to distinguish one’s perspective from others, it is also important in social cognition, role-taking, and communication (Brown, 1977).

### 1.3 Emotional intelligence

Emotions, which are the cognitive equivalent of perception (Ribot, 1897), are generally the responses of the individual to events that are valuable to them. These events can be internal or external, and these emotions can be positive or negative. Emotional intelligence is conceptualized as a dimension of social intelligence encompassing the capacity to perceive, regulate, and differentiate emotions in oneself and others, and to employ this emotional information in shaping cognition and behavior (Salovey and Mayer, 1990; Mayer and Salovey, 1997). Emotional intelligence has four distinct branches, from perceiving and reflecting emotions to regulating them.

Individual differences in the educational process arise from the differences in students’ cognitive and emotional intelligence, learning styles, and strategies. The effects of IQ and EQ on academic success in general and language learning in particular have long been discussed. The affective factors influencing foreign language acquisition have recently attracted the attention of numerous scholars and practitioners (Taheri et al., 2019). Swain (2013) states that emotions are actually part of cognition and that language learning is not only a cognitive but also an emotional process; Chao (2003) posits that emotional intelligence competencies function as a comprehensive determinant of students’ academic performance and language acquisition. Schumann’s (1997) Neurobiology Theory also argues that emotion underlies most, if not all, cognition and that variable success in foreign language acquisition is emotionally driven, while Méndez Lopez (2011) states that emotions in the language learning process provide motivational energy for students to direct their actions and organize their thoughts.

In the process of foreign language education in the classroom environment, students are usually afraid of making mistakes and do not want to show their skills until they think they are perfect. Therefore, emotional factors, especially interpersonal competencies and stress management skills are very important (Pishghadam, 2009). Krashen (2009) says that people can only learn a foreign language with comprehensible language input. He claims that the high emotional filter, which distracts students’ attention when it is high (Du, 2009), prevents language input from reaching the responsible brain part (Krashen, 2009), and that acquisition is inevitable when the emotional filter (Krashen, 1980) decreases and with appropriate comprehensible language input. In this case, in addition to being exposed to meaningful language input, students’ emotional filter status should also be kept under control. The student is likely to adopt a defensive reaction in the presence of any threat of failure. These reactions can be aggressive (Ribot, 1897) or withdrawal, which negatively affects language use (Krashen, 1980). Stevick (1976) states that the more internal a language unit is, the more permanent it will be. However, the criterion that determines internality is the emotional filter. The emotional filter must be reduced enough to allow language input to be received. Otherwise, even if students understand the language input, permanent learning may not occur. This can also negatively affect motivation and cause an inefficient process in language learning.

Foreign language learning is a process that carries many problems and difficulties due to factors such as mother tongue effect, target language features, etc., and because it is a learning outcome rather than an acquisition. In addition to the difficulties brought by this process, another aspect of the problems is that students encounter both cognitive and emotional difficulties and obstacles. Speaking skills are productive and complex by nature. Speaking skills in foreign language learning require not only knowing the target language but also the acquisition of the characteristics of speaking skills. Metacognitive awareness, which is the knowledge of a person’s cognitive process (Flavell, 1976; cited in Brown, 1977), provides the individual with the ability to control their earning status and target-oriented actions. In addition to this difference in ability regarding cognitive processes, students’ feelings during the language learning process are also determinants of their motivation and self-efficacy. The control and order of an individual’s feelings are managed by emotional intelligence (Mayer and Salovey, 1997), which helps the individual understand and regulate emotions. It is known that the defensive learning approach caused by various emotions in particular affects student behaviors to the extent that it can hinder language input. It has been wondered whether these factors are also effective in foreign language speaking skills. Based on this, this study investigated the relationship between students’ levels of being able to control and manage their emotions and metacognitive awareness and their foreign language speaking self-efficacy. Thus, it was tried to determine whether both metacognitive awareness and emotional intelligence affect students’ efficiency in language education. In line with this purpose, the following question was sought in the study:

1. Do emotional intelligence and metacognitive awareness predict foreign language speaking self-efficacy?

## 2 Method

### 2.1 Model

This study adopts an explanatory research design based on the correlational approach. Correlational research, as a type of relational inquiry, examines the associations among two or more variables without implementing any external manipulation. A correlation between two variables indicates that the scores of one variable are systematically related to the scores of the other. A positive correlation signifies that higher scores on one variable correspond to higher scores on the other, and lower scores likewise align. In contrast, a negative correlation demonstrates that higher scores on one variable are linked to lower scores on the other, and lower scores correspond to higher scores (Fraenkel et al., 2012).

### 2.2 Participants

The study group of the research was determined using the purposive sampling method, which is a non-random sampling method. In this sampling method, the researcher’s prior knowledge of the population and the purpose of the study are important. Based on these, the researcher selects a sample based on their personal judgment. Purposive sampling differs from convenience sampling in that people use their judgment to select a sample that they believe will provide the data they need, rather than examining everyone available (Fraenkel et al., 2012). For this study, it was aimed to reach students who are learning Turkish as a foreign language at Bolu Abant İzzet Baysal University, who know enough Turkish to provide the required data, and who continue their education to speak Turkish well. Therefore, those who learn Turkish at the B1 level was thought to be more suitable. The study group of this research consisted of 113 students who were at B1 level and were still learning Turkish at Bolu Abant İzzet Baysal University in the 2024–2025 academic year. All participants were informed through the informed consent form and signed the form. In addition, in correlational studies, the minimum sample size accepted is expected to be at least 30 to avoid giving incorrect estimates of the degree of relationship (Fraenkel et al., 2012). It is also seen that the sample size of this study does not pose a problem in this respect.

### 2.3 Tools

#### 2.3.1 Emotional Intelligence Scale

Adapted to Turkish the Emotional Intelligence Scale, developed by Hyuneung Lee and Yungjung Kwak in South Korea in 2011, was adapted to Turkish by applying it to 249 people. In the confirmatory factor analysis, 3-dimensional models consisting of 20 items were found to be consistent. Internal consistency, item, and factor analysis studies were conducted to examine the psychometric properties of the scale. The reliability results of the scale analyses were 0.83, and the scale was found to be reliable. The confirmatory factor analysis results reveal that the original 3-dimensional models of the scale are suitable for the Turkish sample (χ^2^ = 399.55, df = 167, RMSEA = 0.075, CFI = 0.91, NNFI = 0.90, SRMR = 0.080, IFI = 0.91, GFI = 0.86). In this case, the scale is thought to be a reliable and valid tool for use in the educational process in Türkiye (Kayıhan and Arslan, 2016).

#### 2.3.2 Metacognitive Awareness Inventory

The Metacognitive Awareness Inventory developed by Schraw and Dennison (1994) consists of 52 items. This inventory has a 5-point Likert-type rating of (1) Never (2) Rarely (3) Often (4) Usually and (5) Always. In the study conducted on 607 university students, exploratory factor analysis and convergent validity were applied as construct validity. Internal consistency and test–retest coefficients were examined for reliability. As a result of linguistic equivalence, it was seen that the correlation coefficient between the original and adapted form scores of the scale was 0.89. This result shows that the Turkish translation of the inventory items is similar to the original English items and that the Turkish form and the English form are equivalent (Akın et al., 2007).

#### 2.3.3 Speaking skills self-efficacy scale for students learning Turkish as a foreign language

The scale developed by Kurudayıoğlu and Güngör (2017) is an 11-point Likert-type scale consisting of 17 items. The scale is rated as: *0*: “I Definitely Cannot Do It”; *10, 20, 30, 40, 50*: “I Can Do It at a Moderate Level”; *60, 70, 80, 90, 100*: “I Definitely Can Do It.” The KMO value of the scale is 0.959; the Barlett test result and chi-square test statistics are significant (χ^2^ = 2335.989; SD: 136; *p* = 0.000) and it was determined that the scale was suitable for factor analysis. The item analysis shows that the Cronbach Alpha internal consistency coefficient is 0.951, that is, it is reliable.

### 2.4 Data collection

The study data consisted of scales completed by participants. Data were collected through document review.

### 2.5 Data analysis

The data of the study were analyzed using IBM SPSS Statistics 27. Before proceeding with multiple regression analysis, the assumptions of normality, linearity, homoscedasticity, independence of errors, and multicollinearity were examined. Normality was assessed through histograms of standardized residuals and P-P plots. Linearity and homoscedasticity were evaluated using scatterplots of standardized residuals against predicted values. Independence of errors was examined using the Durbin–Watson statistic, and multicollinearity was assessed through Variance Inflation Factor (VIF) values. Mean scores were calculated for all scales, with higher scores indicating higher levels of the respective constructs. Following these checks, multiple regression analysis was conducted using the enter method to identify the predictors of participants’ self-efficacy in speaking Turkish as a foreign language.

### 2.6 Validity and reliability

The reliability result of the Emotional Intelligence Scale for this research was 0.83 and was found to be reliable; for the reliability result of the Metacognitive Awareness Inventory for this study was 0.92 and was found to be reliable; and the reliability result of the Speaking Skills Self-Efficacy Scale for Students Learning Turkish as a Foreign Language for this study is 0.93 and it was found to be reliable.

## 3 Findings

**Figure 1:**
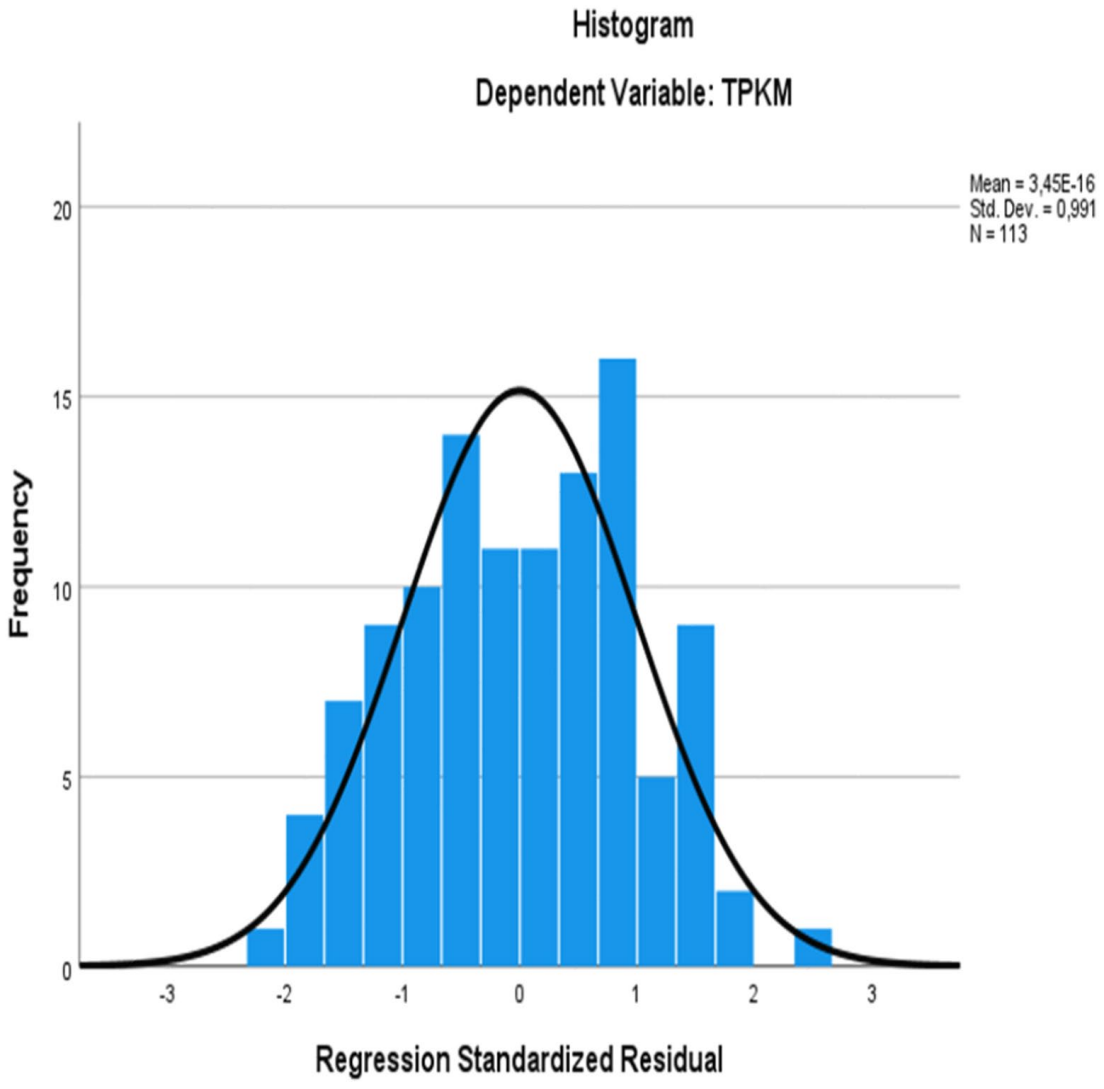
Histogram of normally distributed residuals.

**Figure 2:**
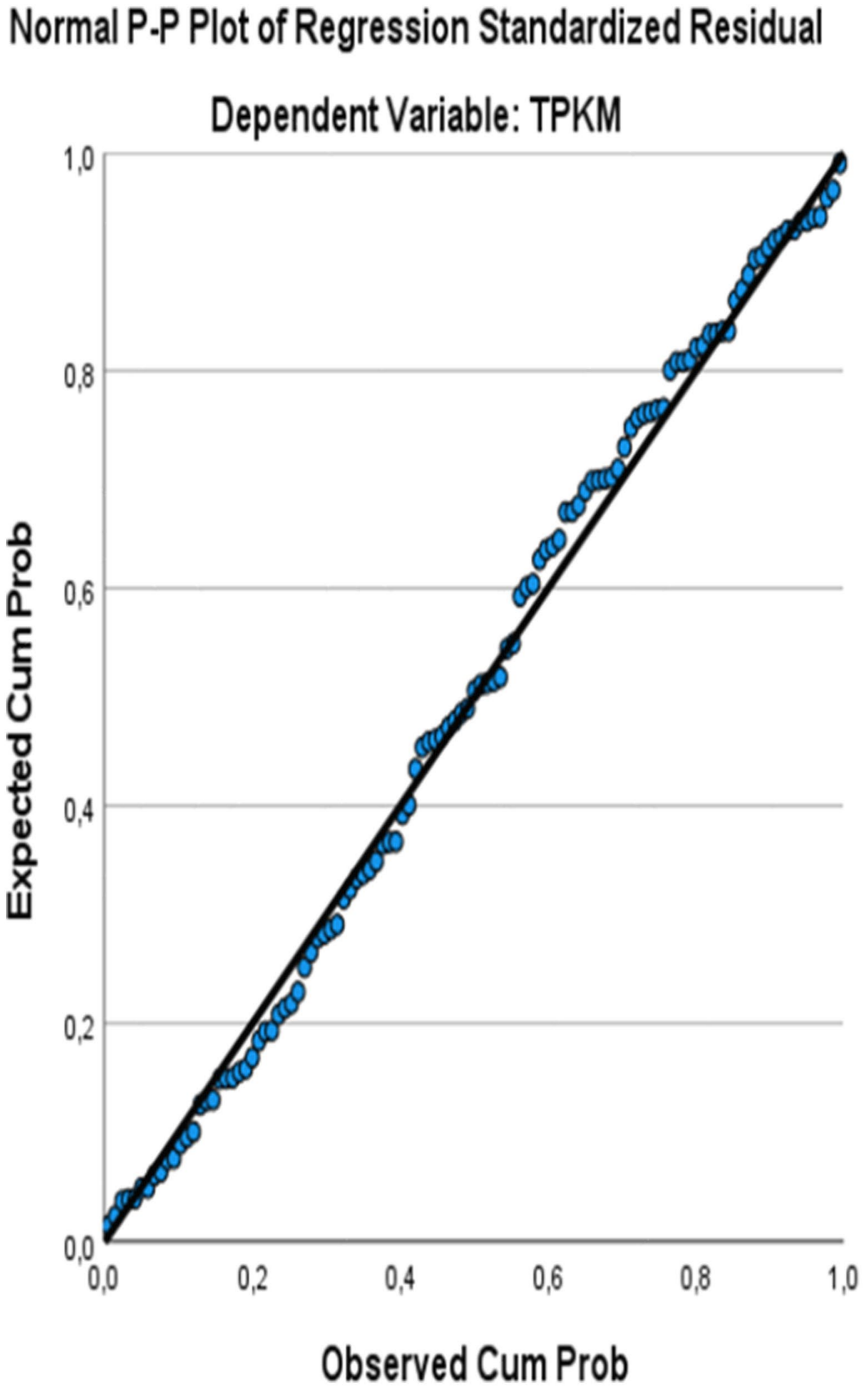
P-P plots of normally distributed residuals.

**Figure 3:**
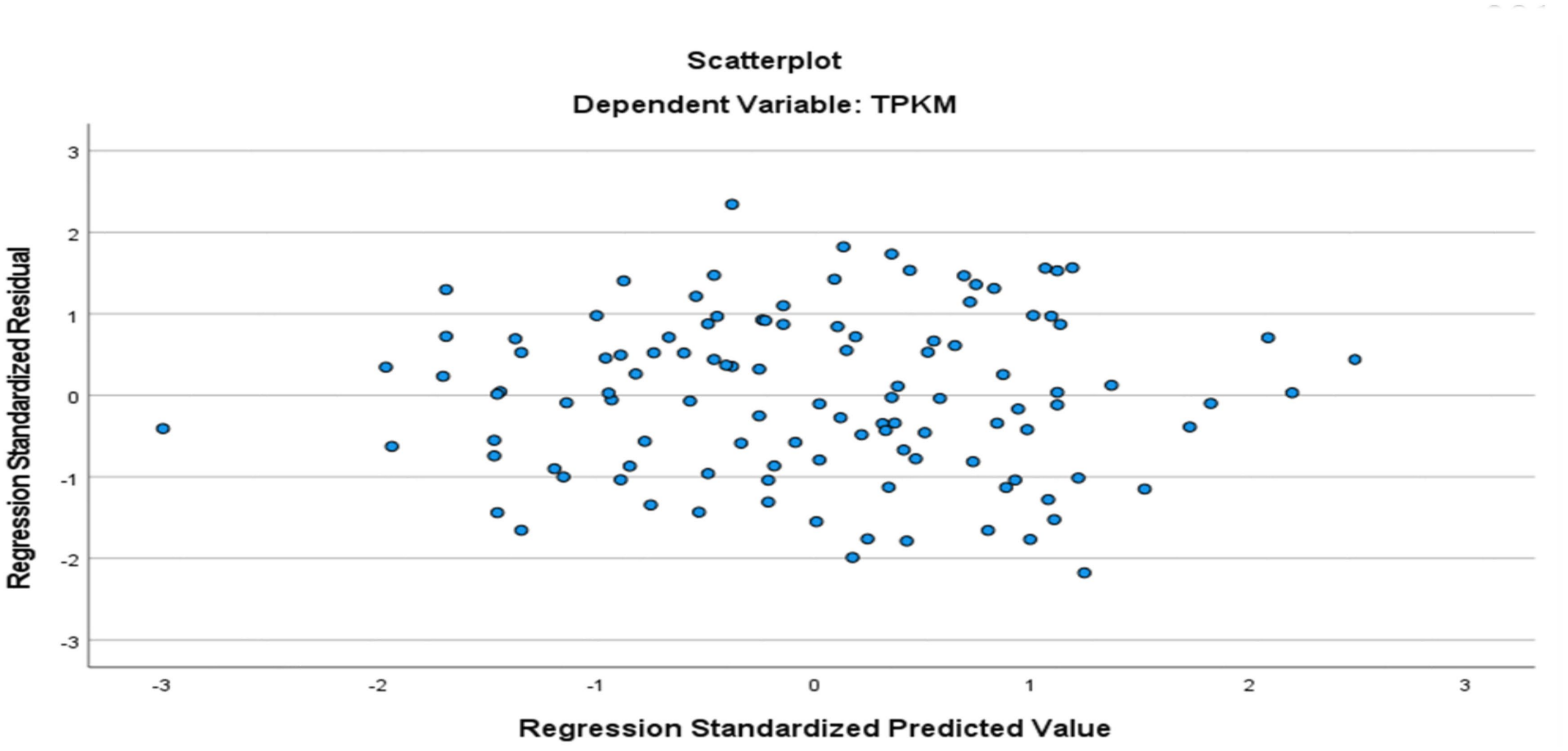
Scatterplot of *ZRESID against ZPRED.

The relation between emotional intelligence and metacognitive awareness and speaking skills self-efficacy of those learning Turkish as a foreign language was determined by multiple regression analysis. Accordingly, multiple linear regression analysis was applied to test the relationship between metacognitive awareness, emotional intelligence and speaking self-efficacy. The multiple linear regression model was created with the enter method, where all independent variables were entered into the model at the same time. The VIF values found as a result of the analysis being less than 10 and close to 1 indicate that there is no multicollinearity problem in the model (Field, 2018). Before interpreting the regression results, the assumptions of multiple linear regression were examined. While the histogram of standardized residuals and the P-P plots (Figures 1, 2) showed an approximately normal distribution, the scatter plot of standardized residuals against predicted values (Figure 3) indicated that the assumptions of linearity and homoskedasticity were met.

The histogram of regression standardized residuals and P-P plot normality assumption was checked. In Figures 1, 2, it is seen that the shape of the histogram is approximately on the normal curve.

The absence of a clear pattern in the scatter plot of the predicted value and the residual (Figure 3) shows that the homoscedasticity assumption is not violated. The value of the Durbin-Watson coefficient is found 1.441. The Durbin-Watson coefficient value being less than 4 also indicates that there is no autocorrelation (Durbin and Watson, 1951) (see Table 1).

**Table 1:** Multiple linear regression table on the determining role of metacognitive awareness and emotional intelligence on speaking self-efficacy.

Variable	B	SE	Beta (β)	*t*	*p*	VIF
Constant	−285	232.978		−1.224	0.223	
Emotional IQ	2.065	2.931	0.069	0.705	0.482	1.425
Metacognitive awareness	6.200	1.334	0.458	4.649	<0.001	1.425
*R* = 0.500	*R^2^* = 0.250	*Adj. R^2^* = 0.236		*F*(2. 110) = 18.296, *p* < 0.001		

The multiple linear regression analysis model was found to be compatible with the data: *F*(2. 110) = 18.296, *p* < 0.001. The model was found to explain 23.6% of the observed variance in the speaking self-efficacy score. As a result of the multiple linear regression analysis, it was revealed that as metacognitive awareness increased, the speaking self-efficacy level increased significantly (*β* = 0.46, *p* < 0.001), whereas the emotional intelligence level did not have a statistically significant effect on speaking self-efficacy (β = 0.07, *p* = 0.482). According to the multiple linear regression model, a 1-point average score increase from the Metacognitive Awareness Inventory, where the average of possible scores is between 1 and 5, statistically significantly predicts an average of 6.200 points higher from the Speaking Self-Efficacy Scale, where the scores can be obtained between 0 and 100. The score obtained from the Emotional Intelligence Scale does not have an explanatory role regarding the score obtained from the Speaking Self-Efficacy Scale.

## 4 Conclusion and discussion

In this study, it was tried to determine whether emotional intelligence and metacognitive awareness have an effect on self-efficacy in speaking in foreign language. Multiple regression analysis was performed for this purpose. While the analysis results showed that there was a significant positive relation between students’ speaking self-efficacy and metacognitive awareness, no statistically significant relation was found between emotional intelligence and speaking self-efficacy. In this case, it can be said that only metacognitive awareness among the independent variables significantly predicts the level of speaking self-efficacy.

The relationship between metacognitive awareness and speaking ability involves cognitive functions such as accessing and controlling information, correcting when necessary, and redesigning. The fact that students’ beliefs about themselves and the target task are positively affected by an awareness that can control themselves cognitively is a result that is compatible with the nature of knowledge. This finding appears theoretically consistent with social cognitive theory, which emphasizes that self-efficacy is shaped by individuals’ perceived ability to regulate task-related processes (Bandura, 1977). Metacognitive awareness may strengthen students’ sense of control and confidence in performing foreign language speaking tasks by enabling them to plan, monitor, and evaluate their own speaking performance. Previous studies on second language learning similarly highlight the role of metacognitive regulation in enhancing self-efficacy beliefs and language performance (Nosratinia et al., 2014; Schunk, 2003; Teng et al., 2023). When we look at the studies in the literature, it is seen that the general self-efficacy of foreign language students is related to metacognitive awareness (Nosratinia et al., 2014); the positive relationship between high self-efficacy and high achievement (Asakereh and Dehghannezhad, 2015; Hermagustiana et al., 2021; Khatib and Maarof, 2015; Suharja, 2020; Wang et al., 2009); it is observed that high self-efficacy enables language learners to face various obstacles in speaking skills (Wijaya, 2024) and the positive relationship between speaking self-efficacy and word order is mentioned (Ocarina et al., 2021).

On the other hand, it was also found that students’ speaking performance and self-efficacy were not related (Givency, 2023). The results of this study are parallel to the positive relationships determined in studies examining the effect of metacognitive awareness on foreign language learning and speaking skills (Adam, 2016; Derakhshan and Fathi, 2024; Safdari and Farzi, 2018; Shin, 2024; Teng et al., 2023). In addition, it was also found in the literature that there was no correlation between metacognitive awareness and success in a foreign language (Doğan and Tuncer, 2017). It is seen that studies on foreign language learning with metacognitive awareness focus more on listening and reading skills.

Another finding of the study is that the emotional intelligence variable is not related to students’ foreign language speaking self-efficacy. Since speaking skills require more complex cognitive processes, emotional intelligence may affect foreign language speaking self-efficacy in more indirect ways or a longer term. The fact that metacognitive awareness provides the individual with the authority to directly evaluate and intervene in his/her cognitive state suggests that self-efficacy is directly related to information control rather than emotions in this study. Emotional intelligence may not be able to control the cognitive skills and competence required for speaking skills. At the same time, self-efficacy consists of resources that can be affected by past experiences and the environment in which they are located. Therefore, these resources of the individual may not have been tolerated by emotional intelligence. The fact that speaking skills can be reinforced with knowledge and experience rather than emotional situations that affect individuals may have paved the way for it not to show a direct relation with emotional intelligence.

Emotional intelligence and its subcomponents generally include features such as recognizing, being aware of, and controlling emotions, and may not be an easily obtained situation. Not having emotional intelligence at a level that can control mediating variables such as anxiety and self-confidence between two variables may also cause a relationship not to be established with emotional intelligence. This study is limited only to speaking skills, and when the literature is examined, studies show that there is a positive, high, and significant relation between emotional intelligence levels and success in a foreign language (Çakıcı, 2017; Ebrahimi et al., 2018; Kurniasih and Retnaningsih, 2018; Nusantara et al., 2021; Soodman Afshar and Rahimi, 2014; Surahman, 2020; Tikupasang and Nasmilah, 2023; Wang et al., 2024). There are also studies indicating that emotional intelligence has a weak effect on speaking performance (Afifah et al., 2024) or that emotional intelligence has no effect on students’ speaking performance (Adelia et al., 2022; Santoso et al., 2024). No study has been found in the literature related to emotional intelligence and self-efficacy in speaking a foreign language.

From a pedagogical perspective, the findings of this study, despite its contextual limitations within the Turkish as a foreign language context, suggest that teaching practices and classroom activities aimed at developing speaking self-efficacy should prioritize the enhancement of learners’ metacognitive awareness. Classroom activities that encourage planning, self-monitoring, and reflective evaluation opportunities may help learners strengthen their self-efficacy beliefs by providing greater control over their speaking performance. Emotional intelligence, while an important individual characteristic in language learning, may influence speaking self-efficacy indirectly through affective variables such as psychological states or classroom interaction patterns. Future research employing longitudinal designs and diverse proficiency levels may further clarify the complex relationships among emotional intelligence, metacognitive awareness, and speaking self-efficacy. One of the limitations of this study, considering the fact that the sample was only Turkish learners at the B1 level, may have affected the results of the study. In future studies, the generalizability of the findings can be increased by examining the relations between emotional intelligence and metacognitive awareness levels of individuals with different language levels and their speaking self-efficacy. In addition, long-term studies may be useful to evaluate the development of emotional intelligence and metacognitive awareness skills over time.

## Data Availability

The raw data supporting the conclusions of this article will be made available by the authors, without undue reservation.

## References

[ref1] Adam A. (2016). Relationship between students’ metacognitive strategy and self-efficacy in speaking. ANGLO-SAXON: Jurnal Ilmiah Program Studi Pendidikan Bahasa Inggris 7, 105–121.

[ref2] Adelia E. Sudirman A. M. Yunus R. Y. I. (2022). The correlation between emotional intelligence and speaking performance. DEIKTIS Jurnal Pendidikan Bahasa dan Sastra 2, 519–528. doi: 10.53769/deiktis.v2i4.374

[ref3] Afifah M. Ningrum A. S. B. Wahyuni S. Syaifulloh B. (2024). Self-efficacy, anxiety, and emotional intelligence: do they contribute to speaking performance? JOLLT J. Lang. Lang. Teach. 12, 793–806. doi: 10.33394/jollt.v12i2.10798

[ref4] Akın A. Abacı R. Çetin B. (2007). Bilişötesi Farkındalık Envanteri’nin Türkçe formunun geçerlik ve güvenirlik çalışması. Kuram ve Uygulamada Eğitim Bilimleri 7, 655–680.

[ref5] Aleksandrzak M. (2011). Problems and challenges in teaching and learning speaking at an advanced level. Glottodidactica. 37, 37–48.

[ref6] Alonso R. A. (2018). “Speaking in a second language. Where are we now?” in Speaking in a second language. ed. Alonso Alonso R. (Amsterdam/Philadelphia: John Benjamins Publishing Company), 225–240.

[ref7] Asakereh A. Dehghannezhad M. (2015). Student satisfaction with EFL speaking classes: relating speaking self-efficacy and skills achievement. Issues Educ. Res. 25, 345–363.

[ref8] Baker L. Brown A. L. (1980). Metacognitive skills and reading. Technical report. Available online at: https://eric.ed.gov/?id=ED195932

[ref9] Bandura A. (1977). Self-efficacy: toward a unifying theory a behavioral change. Psychol. Rev. 84, 191–215, 847061 10.1037//0033-295x.84.2.191

[ref10] Bandura A. (1982). Self-efficacy mechanism in human agency. Am. Psychol. 37, 122–147. doi: 10.1037/0003-066x.37.2.122

[ref11] Birdsong D. (2009). “Second language acquisition and the critical period hypothesis” in Whys and why nots of the critical period hypothesis for second language acquisition. ed. Birdsong D. (London, UK: Taylor & Francis e-Library), 1–22.

[ref12] Brown L. (1977). Knowing when, where, and now to remember: a problem of metacognition. In Glaser R.. Advences in instructional psychology. Available online at: https://files.eric.ed.gov/fulltext/ED146562.pdf

[ref13] Brown G. Yule G. 1983 Teaching the spoken language Cambridge University Press Available online at: https://assets.cambridge.org/97805212/73848/excerpt/9780521273848_excerpt.pdf

[ref14] Çakıcı D. (2017). Duygusal zekâ ve yabancı dil başarısı arasındaki ilişki üzerine bir çalışma. Int. J. Lang. Educ. Teach. 5, 94–104. doi: 10.18298/ijlet.1754

[ref15] Castro O. Peck V. (2005). Learning styles and foreign language learning difficulties. Foreign Lang. Ann. 38, 401–409. doi: 10.1111/j.1944-9720.2005.tb02226.x

[ref16] Chao C. T. (2003). Foreign language anxiety and emotional intelligence: A study of EFL students in Taiwan. Kingsville, TX, USA: Texas A&M University-Kingsville.

[ref17] Chen T. Y. Chang G. B. (2004). The relationship between foreign language anxiety and learning difficulties. Foreign Lang. Ann. 37, 279–289.

[ref18] de Vries H. Dijkstra M. Kuhlman P. (1988). Self-efficacy: the third factor besides attitude and subjective norm as a predictor of behavioural intentions. Health Educ. Res. 3, 273–282. doi: 10.1093/her/3.3.273

[ref19] Derakhshan A. Fathi J. (2024). Growth mindset, self-efficacy, and self-regulation: a symphony of success in L2 speaking. System 123:103320. doi: 10.1016/j.system.2024.103320

[ref20] Doğan Y. Tuncer M. (2017). Effect of metacognitive awareness on achievement in foreign language learning. Istanbul Comm. Univ. J. Soc. Sci. 16, 297–310.

[ref21] Du X. (2009). The affective filter in second language teaching. Asian Soc. Sci. 5, 162–165. doi: 10.5539/ass.v5n8p162

[ref22] Durbin J. Watson G. S. (1951). Testing for serial correlation in least squares regression II. Biometrika 38, 159–178. doi: 10.1093/biomet/38.1-2.159 14848121

[ref23] Ebrahimi M. R. Khoshsima H. Zare-Behtash E. Heydarnejad T. (2018). Emotional intelligence enhancement impacts on developing speaking skill among EFL learners: an empirical study. Int. J. Instr. 11, 625–640. doi: 10.12973/iji.2018.11439a

[ref24] Field A. P. (2018). Discovering statistics using IBM SPSS statistics. 5th Edn. London, UK: Sage.

[ref25] Flege J. E. (1997). English vowel production by Dutch talkers: more evidence for the “similar” vs. “new” distinction. Second-languagespeech. Structure and process. eds. James A. Leather J. (NY: Mouton de Gruyter), 11–52.

[ref26] Fraenkel J. Wallen N. Hyun H. (2012). How to design and evaluate education research. Eight Edn. New York, NY, USA: McGraw-Hill.

[ref27] Fulcher G. (2014). Testing second language speaking. London, UK: Routledge.

[ref28] Ganschow L. Sparks R. L. Javorsky J. (1998). Foreign language learning difficulties: an historical perspective. J. Learn. Disabil. 31, 248–258. doi: 10.1177/002221949803100304 9599957

[ref29] Ganschow L. Sparks R. Scheider E. (1995). Learning a foreign language: challenges for students with language learning difficulties. Dyslexia 1, 75–95.

[ref30] Gilakjani A. Ahmadi M. (2011). A study of factors affecting EFL learners’ comprehension and strategies for improvement. J. Lang. Teach. Res. 2, 977–988. doi: 10.4304/jltr.2.5.977-988

[ref31] Givency C. (2023). The correlation between self-efficacy and speaking performance of the eleventh-grade students at SMAN 1 Palangka Raya. EBONY: J. English Lang. Teach. Ling. Lit. 3, 139–150. doi: 10.37304/ebony.v3i2.8599

[ref32] Hermagustiana I. Astuti A. D. Sucahyo D. (2021). Do I speak anxiously? A correlation of self-efficacy, foreign language learning anxiety and speaking performance. Script J. 6, 68–80. doi: 10.24903/sj.v6i1.696

[ref33] Horwitz E. K. Horwitz M. B. Cope J. (1986). Foreign language classroom anxiety. Mod. Lang. J. 70, 125–132.

[ref34] Jabbarifar T. (2011). The importance of self-efficacy and foreign language learning in the 21st century. J. Int. Educ. Res. 7:117. doi: 10.19030/jier.v7i4.6196

[ref35] Kayıhan Ş. N. Arslan S. (2016). Emotional intelligence scale: a study of scale adaptation. FSM İlmi Araştırmalar İnsan ve Toplum Bilimler Dergisi 7, 137–145.

[ref36] Khatib F. M. M. Maarof N. (2015). Self-efficacy perception of oral communication ability among English as a second language (ESL) technical students. PRO 204, 98–104. doi: 10.1016/j.sbspro.2015.08.121

[ref37] Krashen S. D. (1980). “The input hypothesis” in Current issues in bilingual education. ed. Alatis J. E. (New York, NY, USA: Longman), 168–180.

[ref38] Krashen S. D. (2002). Second language aqcusition and second language learning (Internet edition). Los Angeles, CA, USA: University of California.

[ref39] Krashen S. D. (2009). Principles and practice in second language acquisition. İnternet Edn. Los Angeles, CA, USA: University of Southern California.

[ref40] Kurniasih N. Retnaningsih W. 2018 The correlation between emotional quotient (EQ), self—confidence, and speaking ability at the eighth grade students of Smp N 2 Gombong Kebumen in the academic year 2017/2018. [Skripsi thesis]. Surakarta, Indonesia: IAIN Surakarta.

[ref41] Kurudayıoğlu M. ve Güngör H. (2017). Yabancı dil olarak Türkçe öğrenenlerin konuşma öz yeterliklerinin çeşitli değişkenler açısından incelenmesi. Uluslararası Türkçe Edebiyat Kültür Eğitim Dergisi, 6, 1105–1121. Available online at: https://dergipark.org.tr/tr/pub/teke/issue/30306/327518.

[ref42] Lane J. Lane A. M. Kyprianou A. (2004). Self-efficacy, self-esteem and their impact on academic performance. Soc. Behav. Pers. 32, 247–256. doi: 10.2224/sbp.2004.32.3.247

[ref43] Lenneberg E. H. (1967). The biological foundations of language. Hosp. Pract. 2, 59–67. doi: 10.1080/21548331.1967.11707799

[ref44] Linnenbrink E. Pintrich P. (2003). The role of self efficacy beliefs instudent engagement and learning intheclassroom. Read. Writ. Q. 19, 119–137. doi: 10.1080/10573560308223

[ref45] Lisnawati I. Yuniawati Y. Kartadireja W. N. (2019). Student’s self-efficacy in speaking learning. In international symposium on social sciences, education, and humanities (ISSEH 2018). Paris, France: Atlantis Press, 255–261.

[ref46] Lowie W. Verspoor M. van Dijk M. (2018). “The acquisition of L2 speaking a dynamic perspective” in Speaking in a second language. ed. Alonso R. A. (Amsterdam/Philadelphia: John Benjamins Publishing Company).

[ref47] Ma Y. (2022). The triarchy of L2 learners’ emotion, cognition, and language performance: anxiety, self-efficacy, and speaking skill in lights of the emerging theories in SLA. Front. Psychol. 13:1002492. doi: 10.3389/fpsyg.2022.1002492, 36204743 PMC9530130

[ref48] Mayer J. D. Salovey P. (1997). “What is emotional intelligence?” in Emotional development and emotional intelligence: Educational implications. eds. Salovey P. Sluyter D. J. (New York, NY, USA: Basic Books), 3–34.

[ref49] Méndez Lopez M. G. (2011). The motivational properties of emotions in foreign language learning. Colomb. Appl. Linguist. J. 13, 43–58. doi: 10.14483/22487085.3764

[ref50] Metcalfe J. (2008). “Evolution of metacognition” in Handbook of metamemory and memory, RBjork. ed. Dunlosky J. (Hove, UK: Psychol. Press), 29–46.

[ref51] Munro J. M. (2008). “Foreign accent and speech intelligibility” in Phonology and second language acquisition. eds. Hansen Edwards J. Zampini M. L. (Amsterdam/Philadelphia: John Benjamin Publishing), 193–218.

[ref52] Nickel G. (1971). Problems of learners' difficulties in foreign language acquisition. Int. Rev. Appl. Linguist. Lang. Teach. 9. doi: 10.1515/iral.1971.9.3.219

[ref53] Nosratinia M. Saveiy M. Zaker A. (2014). EFL learners' self-efficacy, metacognitive awareness, and use of language learning strategies: how are they associated. Theory Pract. Lang. Stud. 4, 1080–1092. doi: 10.4304/tpls.4.5.1080-1092

[ref54] Nusantara T. B. Rahman M. A. Mahmud M. (2021). Students’ emotional intelligence, self-efficacy, and schemata in speaking performance. Celebes J. Lang. Stud. 1, 166–184. doi: 10.51629/cjls.v1i2.59

[ref55] Ocarina D. Anwar K. Marifah U. (2021). The correlation between students’ speaking self-efficacy and collocation competence in speaking at SMPN 1 Parengan. J. Engl. Teach. Lit. Appl. Linguist. 5, 101–1108. doi: 10.30587/jetlal.v5i2.3742

[ref56] Oxford R. Shearin J. (1994). Language learning motivation: expanding the theoretical framework. Mod. Lang. J. 78, 12–27.

[ref57] Pajares F. (1996). Self-efficacy beliefs in academic settings. Rev. Educ. Res. 66, 543–578. doi: 10.3102/00346543066004543

[ref58] Paris S. G. Winograd P. (2013). “How metacognition can promote academic learning and instruction” in Dimensions of thinking and cognitive instruction (London, UK: Routledge), 15–51.

[ref59] Penfield W. Roberts L. (1959). Speech and brain mechanisms. Princeton, NJ, USA: Princeton University Press.

[ref60] Piechurska-Kuciel E. (2014). Self-efficacy in the foreign language learning process: the language anxiety perspective. Linguist. Siles. 35, 397–415.

[ref61] Pishghadam R. (2009). A quantitative analysis of the relationship between emotional intelligence and foreign language learning. Electron. J. Foreign Lang. Teach. 6, 31–41.

[ref62] Raoofi S. Tan B. H. Chan S. H. (2012). Self-efficacy in second/foreign language learning contexts. Engl. Lang. Teach. 5, 60–73. doi: 10.5539/elt.v5n11p60

[ref63] Ribot Th. (1897). The psychology of the emotions, the contemporary science series. (Ed.) Ellis Havelock. Available online at: http://www.archive.org/details/psychologyofemoOOribo.

[ref64] Safdari S. Farzi S. (2018). Enhancing EFL learners’ self efficacy beliefs through raising metacognitive awareness. J. Teach. Engl. Lang. Stud. 6, 144–163.

[ref65] Salovey P. Mayer J. D. (1990). Emotional intelligence. Imagination Cogn. Pers. 9, 185–211. doi: 10.2190/DUGG-P24E-52WK-6CDG

[ref66] Santoso D. R. Affandi G. R. Basthomi Y. (2024). ‘Getting stuck’: a study of Indonesian EFL learners’ self-efficacy, emotional intelligence, and speaking achievement. Stud. Engl. Lang. Educ. 11, 384–402. doi: 10.24815/siele.v11i1.30969

[ref9001] Schraw G. Dennison R. S. (1994). Assessing metacognitive awareness. Contemp. Educ. Psychol. 19, 460–475. doi: 10.1006/ceps.1994.1033

[ref67] Schumann J. H. (1997). The neurobiology of affect in language by John H. Schumann. Oxford, UK: Blackwell Publishers, 341.

[ref68] Schunk D. H. (2003). Self-efficacy for reading and writing: influence of modeling, goal setting, and self-evaluation. Read. Writ. Q. 19, 159–172. doi: 10.1080/10573560308219

[ref69] Shin M. H. (2024). Improving English speaking skills in a college general English course using metacognitive strategies. Engl. Teach. 79, 99–121. doi: 10.15858/engtea.79.3.202409.99

[ref70] Soodman Afshar H. Rahimi M. (2014). The relationship among emotional intelligence, critical thinking, and speaking ability of Iranian EFL learners. Teach. Engl. Lang. 8, 31–59. doi: 10.22132/tel.2014.54564

[ref71] Stam G. (2018). “Gesture and speaking a second language” in Speaking in a second language. ed. Alonso Alonso R. (Amsterdam/Philadelphia: John Benjamins Publishing Company).

[ref72] Stevick E. W. (1976). Memory, meaning & method: Some psychological perspectives on language learning. Rowley, MA, USA: Newbury House Publishers.

[ref73] Suharja S. (2020). The correlation of self-efficacy to the students ‘speaking performance in EFL context at University of Dayanu Ikhsanuddin Baubau. Engl. Educ. J., 17–25.

[ref74] Surahman D. (2020). The effect of community language learning and emotional intelligence on students’ speaking skill. Jakarta, Indonesia: State Islamic University (Uin) Syarif Hidayatullah Jakarta.

[ref75] Swain M. (2013). The inseparability of cognition and emotion in second language learning. Lang. Teach. 46, 195–207. doi: 10.1017/S0261444811000486

[ref76] Taheri H. Sadighi F. Bagheri M. S. Bavali M. (2019). EFL learners’ L2 achievement and its relationship with cognitive intelligence, emotional intelligence, learning styles, and language learning strategies. Cogent Educ. 6, 1–21. doi: 10.1080/2331186X.2019.165588

[ref77] Teng M. F. Yang Z. (2023). Metacognition, motivation, self-efficacy belief, and English learning achievement in online learning: longitudinal mediation modeling approach. Innov. Lang. Learn. Teach. 17, 778–794. doi: 10.1080/17501229.2022.2144327

[ref78] Tikupasang O. Nasmilah A. P. (2023). Students’ emotional intelligence and self-efficacy on the speaking performance in politeknik kesehatan mimika. J. Reattach. Ther. Dev. Divers. 6, 1349–1358.

[ref79] Tremblay P. F. Robert C. G. (1995). Expanding motivation construct in language learning. Mod. Lang. J. 79, 505–518.

[ref80] Tschannen-Moran M. Woolfolk Hoy A. Hoy W. K. (1998). Teacher efficacy: its meaning and measure. Rev. Educ. Res. 68, 202–248. doi: 10.3102/00346543068002202

[ref81] Tuan N. H. Mai T. N. (2015). Factors affecting students’ speaking performance at Le Thanh Hien high school. Asian J. Educ. Res. 3, 8–23.

[ref82] Vaidya S. R. (1999). Metacognitive learning strategies for students with learning disabilities. Education 120, 186–186.

[ref83] van Dinther M. Dochy F. Segers M. (2011). Factors affecting students’ self-efficacy in higher education. Educ. Res. Rev. 6, 95–108. doi: 10.1016/j.edurev.2010.10.003

[ref84] Wade S. E. Reynolds R. E. (1989). Developing metacognitive awareness. J. Read. 33, 6–14.

[ref85] Wang W. Rezaei Y. M. Izadpanah S. (2024). Speaking accuracy and fluency among EFL learners: the role of creative thinking, emotional intelligence, and academic enthusiasm. Heliyon 10. doi: 10.1016/j.heliyon.2024.e37620, 39328575 PMC11425133

[ref86] Wang J. Spencer K. Xing M. (2009). Metacognitive beliefs and strategies in learning Chinese as a foreign language. System 37, 46–56. doi: 10.1016/j.system.2008.05.001

[ref87] Wijaya K. (2024). The impacts of self-efficacy on EFL learners’ speaking skills. J. Educ. Lang. Innov. Appl. Linguist. 3, 121–133. doi: 10.37058/jelita.v3i2.6878

[ref88] Yang N. D. (1999). The relationship between EFL learners' beliefs and learning strategy use. System 27, 515–535. doi: 10.1016/s0346-251x(99)00048-2

